# Effect of food intake on 92 neurological biomarkers in plasma

**DOI:** 10.1002/brb3.747

**Published:** 2017-08-01

**Authors:** Magnus Dencker, Ola Björgell, Joanna Hlebowicz

**Affiliations:** ^1^ Department of Medical Imaging and Physiology Skåne University Hospital Lund University Malmö Sweden; ^2^ Department of Clinical Sciences Division of Medicine Skåne University Hospital Lund University Malmö Sweden

**Keywords:** Olink, Proseek Multiplex Neurology I

## Abstract

**Objective:**

This study evaluates the effect of food intake on 92 neurological biomarkers in plasma. Moreover, it investigated if any of the biomarkers were correlated with body mass index.

**Materials and Methods:**

Twenty‐two healthy subjects (11 male and 11 female aged 25.9 ± 4.2 years) were investigated. A total of 92 biomarkers were measured before a standardized meal as well as 30 and 120 min afterward with the Proseek Multiplex Neurology I kit.

**Results:**

The levels for 13 biomarkers decreased significantly (*p* < .001) 30 min after food intake. The levels for four biomarkers remained significantly decreased (*p* < .001) 120 min after food intake. One biomarker increased significantly (*p* < .001) 30 min after food intake. The changes were between 1% and 12%, with an average difference of about 5%. Only one biomarker showed a difference over 10% due to food intake. The biggest difference was observed for Plexin‐B3 120 min after food intake (12%). Of all the 92 neurological biomarkers, only one was correlated with BMI, Kynureninase *r* = .46, *p* < .05.

**Conclusions:**

This study shows that food intake has a very modest effect on 92 different neurological biomarkers. Timing of blood sampling in relation to food intake, therefore, appears not to be a major concern. Only Kynureninase was correlated with BMI. Further studies are warranted in older healthy subjects and in patients with various neurological diseases to determine whether the findings are reproducible in such populations.

## 1 INTRODUCTION

Neurological disease is a major cause of death and disability in the world (Global Burden of Disease Study 2013 Collaborators, 2015). There is a continuous search for novel biomarkers that could improve the assessment in neurological diseases (Lind et al., 2015; Lind, Emami Khoonsari et al., 2016; Lind, Wu et al., 2016; Miclescu, Svahn, & Gordh, 2015; Moen et al., 2016). Digestion of food is known to have significant hemodynamic and metabolic effects (Dencker, Björgell, & Hlebowicz, 2011; Dieden, Gårdinger, Bjorgell, Hlebowicz, & Dencker, 2016; Gårdinger, Bjorgell, Hlebowicz, & Dencker, 2014; Hlebowicz, Lindstedt‐Ingemansson, Björgell, & Dencker, 2011a; Hlebowicz, Lindstedt‐Ingemansson, Björgell, & Dencker, 2011b; Quatela, Callister, Patterson, & MacDonald‐Wicks, 2016; Stensel, 2010), and may therefore affect different biomarkers. It is relevant from a practical point of view to investigate if these biomarkers are affected by food intake, as it would affect sample collection. It could also be of physiological interest to investigate biological responses to food intake. This study evaluates the effect of food intake, in healthy volunteers, on 92 different emerging neurological biomarkers. Moreover, we also investigated if any of the biomarkers were correlated with body mass index. This has, to the best our knowledge, not been done before.

## 2 MATERIALS AND METHODS

### 2.1 Study Population

The trial is registered in the US National Library of Medicine with the trial registration number NCT01027507. The study investigated 22 healthy Caucasians (11 male and 11 female aged 25.9 ± 4.2 years). None of the subjects had a prior history or showed any symptoms of cardiovascular disease or any other chronic diseases. None of the subjects were taking any cardiovascular medication. Standard height and weight were measured and body mass index (BMI) was calculated. The subjects were examined between 7.30 and 11.00 a.m. after an 8‐hr fast. The subjects ingested a standardized meal consisting of 300 g rice pudding (AXA Goda Gröten Risgrynsgröt; Lantmännen AXA, Järna, Sweden). The total caloric value of the meal was 330 kcal: 10% from protein (9 g), 58% from carbohydrates (48 g), and 32% from fat (12 g). Informed consent was obtained from each participant. The study was approved by the regional ethical review board in Lund, Sweden.

### 2.2 Blood samples

Plasma samples were collected in EDTA test tubes before a meal, and 30 min and 120 min after the meal and frozen. No beverages were consumed during the experiment. One of the blood samples collected 30 min after the meal was defective, and therefore excluded from the analysis. The 92 biomarkers were analyzed at the Olink laboratory in Uppsala by the Proximity Extension Assay technique using the Proseek Multiplex Multiplex Neurology I 96 × 96 reagents kit (Olink Bioscience, Uppsala, Sweden), as previously described (Assarsson et al., 2014; Lundberg, Eriksson, Tran, Assarsson, & Fredriksson, 2011). Data are presented as arbitrary units (AU). Values can be transformed to actual concentrations using transformation algorithms on the Olink Bioscience website (www.olink.com). The conversion, however, is not exact. The 92 biomarkers that were analyzed are as follows (intra‐assay and inter‐assay variation, by manufacturer data (Olink Bioscience, Uppsala, Sweden)): Nicotinamide/nicotinic acid mononucleotide adenylyltransferase 1 (9%,5%), Neuropilin‐2 (6%,5%), Brain‐derived neurotrophic factor (9%,9%), Cell adhesion molecule 3 (9%,7%), Glial cell line‐derived neurotrophic factor (9%,9%), Netrin receptor UNC5C (6%,9%), Brorin (7%,7%), Sialic acid‐binding Ig‐like lectin 9 (5%,7%), CMRF35‐like molecule 6 (7%,7%), Ezrin (7%,10%), SPARC‐related modular calcium‐binding protein 2 (9%,11%), Neuroblastoma suppressor of tumorigenicity 1 (4%,4%), Ephrin‐A4 (9%,10%), Lysosome membrane protein 2 (6%,9%), Neurocan core protein (6%,7%), Protogenin (7%,8%), Roundabout homolog 2 (8%,11%), Cytotoxic and regulatory T‐cell molecule (6%,8%), Repulsive guidance molecule A (6%,9%), Plexin‐B3 (7%,7%), Carboxypeptidase A2 (7%,9%), ADP‐ribosyl cyclase/cyclic ADP‐ribose hydrolase 1 (7%,8%), Sphingomyelin phosphodiesterase (6%,6%), Macrophage scavenger receptor types I and II (6%,5%), Alpha‐2‐macroglobulin receptor‐associated protein (9%,9%), Secreted frizzled‐related protein 3 (9%,8%), Ephrin type‐B receptor 6 (8%,7%), RGM domain family member B (8%,11%), Sialoadhesin (6%,9%), Contactin‐5 (7%,9%), Disintegrin and metalloproteinase domain‐containing protein 22 (7%,7%), C‐type lectin domain family 1 member B (9%,11%), Disintegrin and metalloproteinase domain‐containing protein 23 (7%,12%), Matrilin‐3 (8%,5%), R‐spondin‐1 (9%,12%), Hydroxyacylglutathione hydrolase, mitochondrial (8%,12%), Latexin (9%,7%), Galectin‐8 (7%,7%), Brevican core protein (8%,8%), Layilin (9%,6%), Neprilysin (6%,5%), Growth/differentiation factor 8 (6%,7%), Thy‐1 membrane glycoprotein (6%,9%), WAP, Kazal, immunoglobulin, Kunitz and NTR domain‐containing protein 1 (6%,9%), Transmembrane protease serine 5 (6%,9%), Cadherin‐3 (7%,5%), GDNF family receptor alpha‐1 (7%,12%), Granulocyte‐macrophage colony‐stimulating factor receptor subunit alpha (6%,5%), Beta‐nerve growth factor (8%,7%), Scavenger receptor class A member 5 (6%,10%), OX‐2 membrane glycoprotein (7%,8%), BDNF/NT‐3 growth factors receptor (6%,9%), Granzyme A (8%,7%), Granulocyte Colony‐Stimulating Factor (6%,10%), Draxin (9%,10%), Scavenger receptor class F member 2 (7%,5%), GDNF family receptor alpha‐3 (8%,7%), Poliovirus receptor (6%,8%), Tumor necrosis factor receptor superfamily member 12A (6%,10%), Serine/threonine‐protein kinase receptor R3 (8%,6%), Leucine‐rich repeat transmembrane protein FLRT2 (6%,7%), Carboxypeptidase M (6%,8%), C‐type lectin domain family 10 member A (7%,9%), Glypican‐5 (8%,10%), Bone morphogenetic protein 4 (6%,6%), Fc receptor‐like protein 2 (7%,9%), MAM domain‐containing glycosylphosphatidylinositol anchor protein 1 (9%,14%), Interleukin‐5 receptor subunit alpha (8%,7%), Platelet‐derived growth factor receptor alpha (7%,8%), Dipeptidyl peptidase 1 (5%,10%), Cadherin‐6 (5%,5%), Epithelial discoidin domain‐containing receptor 1 (6%,7%), Junctional adhesion molecule B (8%,8%), Cathepsin S (5%,5%), Neutral ceramidase (5%,12%), N‐acylethanolamine‐hydrolyzing acid amidase (8%,7%), NKG2D ligand 2 (8%,9%), Plexin‐B1 (7%,8%), Tumor necrosis factor receptor superfamily member 21 (7%,10%), CMRF35‐like molecule 1 (8%,9%), Testican‐1 (9%,10%), Interleukin‐12 subunit beta, Interleukin‐12 subunit alpha (7%,5%), Dickkopf‐related protein 4 (9%,9%), Tumor necrosis factor receptor superfamily member 27 (8%,6%), Linker for activation of T‐cells family member 1 (7%,17%), NT‐3 growth factor receptor (6%,11%), Leukocyte‐associated immunoglobulin‐like receptor 2 (7%,7%), Mesencephalic astrocyte‐derived neurotrophic factor (9%,12%), Tenascin‐R (7%,8%), Cell surface glycoprotein CD200 receptor 1 (6%,9%), Neuronal cell adhesion molecule (5%,8%), Kynureninase (7%,8%). A total of seven samples for Brain‐derived neurotrophic factor and one sample for Glial cell line‐derived neurotrophic factor failed to reach detection levels. In these cases, the values were set at the detection levels.

### 2.3 Statistical analysis

Data are presented as mean ± standard deviation (*SD*). Statistical analyses were carried out using Statistica 12 (StatSoft Inc, Tulsa, OK, USA). Comparison between fasting values versus 30 and 120 min after food intake for any given biomarker was analyzed for significance with the Wilcoxon matched pairs test. Univariate relationships between BMI and the 92 neurological biomarkers were assessed by Pearson correlation analysis. Statistical significance was set at *p* < .001 to counteract the problem of multiple comparisons.

## 3 RESULTS

Table 1 displays descriptive statistics of the study population. The levels for 13 biomarkers decreased significantly (*p* < .001) 30 min after food intake. The levels for four biomarkers remained significantly decreased (*p* < .001) 120 min after food intake. One biomarker significantly increased (*p* < .001) 30 min after food intake. The changes were between 1% and 12%, with an average difference of about 5%. Only one biomarker showed a difference over 10% due to food intake. Those biomarkers that were decreased 30 min after food intake include: Granulocyte‐macrophage colony‐stimulating factor receptor subunit alpha (1%), Cathepsin S (2%), Platelet‐derived growth factor receptor alpha (3%), Glypican‐5 (3%), Granzyme A (4%), C‐type lectin domain family 1 member B (4%), CMRF35‐like molecule 1 (4%), Mesencephalic astrocyte‐derived neurotrophic factor (5%), Galectin‐8 (5%), Neuropilin‐2 (5%), Nicotinamide/nicotinic acid mononucleotide adenylyltransferase 1 (7%), and Plexin‐B1 (8%), and Plexin‐B3 (9%). One biomarker that was increased 30 min after food intake was Bone morphogenetic protein 4 (9%). Those biomarkers that were decreased 120 min after food intake include: C‐type lectin domain family 1 member B (5%), Galectin‐8 (7%), Mesencephalic astrocyte‐derived neurotrophic factor (9%), and the biggest difference was observed for Plexin‐B3 (12%). Summary of the findings are in Table 2. Of all the 92 neurological biomarkers, only one was correlated with BMI, Kynureninase *r* = .46, *p* < .05. All other biomarkers had no significant relationship with BMI (data not shown).

**Table 1:** Subjects’ anthropometric characteristics. Values are mean ± *SD*

Variable	
Sex (male/female)	11/11
Body mass (kg)	69 ± 10
Height (cm)	177 ± 8
BMI (kg/m^2^)	21.8 ± 2.2
BSA (m^2^)	1.8 ± 0.2

BMI, Body mass index; BSA, Body surface area.

**Table 2:** Summary of findings for 92 biomarkers before, and 30 and 120 min after a standardized meal. All values are in arbitrary units (Mean ± *SD*)

Variable	Fasting (*n* = 22)	30 min after food intake (*n* = 21)	120 min after food intake (*n* = 22)
Nicotinamide/nicotinic acid mononucleotide adenylyltransferase 1	4.35 ± 1.05	4.03 ± 1.10^a^	3.83 ± 1.09
Neuropilin‐2	3.73 ± 0.40	3.54 ± 0.44^a^	3.64 ± 0.47
Brain‐derived neurotrophic factor	4.16 ± 4.06	4.24 ± 4.14	4.23 ± 4.13
Cell adhesion molecule 3	1.82 ± 0.58	1.84 ± 0.54	1.80 ± 0.61
Glial cell line‐derived neurotrophic factor	0.34 ± 0.30	0.28 ± 0.36	0.45 ± 0.41
Netrin receptor UNC5C	2.61 ± 0.35	2.51 ± 0.35	2.56 ± 0.42
Brorin	3.44 ± 0.57	3.39 ± 0.57	3.37 ± 0.55
Sialic acid‐binding Ig‐like lectin 9	4.30 ± 0.24	4.25 ± 0.28	4.25 ± 0.25
CMRF35‐like molecule 6	4.77 ± 0.30	4.71 ± 0.27	4.73 ± 0.39
Ezrin	4.83 ± 0.32	4.72 ± 0.22	4.63 ± 0.18
SPARC‐related modular calcium‐binding protein 2	8.13 ± 0.55	8.13 ± 0.48	8.08 ± 0.53
Neuroblastoma suppressor of tumorigenicity 1	4.27 ± 0.17	4.30 ± 0.15	4.26 ± 0.15
Ephrin‐A4	2.89 ± 0.30	2.88 ± 0.31	2.79 ± 0.43
Lysosome membrane protein 2	2.17 ± 0.25	2.01 ± 0.33	1.91 ± 0.43
Neurocan core protein	6.90 ± 0.29	6.89 ± 0.33	6.84 ± 0.35
Protogenin	5.33 ± 0.34	5.27 ± 0.32	5.28 ± 0.35
Roundabout homolog 2	4.39 ± 0.36	4.29 ± 0.34	4.38 ± 0.42
Cytotoxic and regulatory T‐cell molecule	3.86 ± 0.48	3.79 ± 0.46	3.87 ± 0.49
Repulsive guidance molecule A	7.69 ± 0.36	7.73 ± 0.35	7.71 ± 0.29
Plexin‐B3	4.84 ± 0.44	4.41 ± 0.42^a^	4.23 ± 0.38^a^
Carboxypeptidase A2	9.06 ± 0.62	9.07 ± 0.55	9.45 ± 0.51
ADP‐ribosyl cyclase/cyclic ADP‐ribose hydrolase 1	3.15 ± 0.53	3.17 ± 0.55	3.01 ± 0.68
Sphingomyelin phosphodiesterase	3.52 ± 0.33	3.45 ± 0.38	3.51 ± 0.40
Macrophage scavenger receptor types I and II	3.79 ± 0.43	3.69 ± 0.43	3.72 ± 0.54
Alpha‐2‐macroglobulin receptor‐associated protein	7.13 ± 0.39	6.95 ± 0.47	7.02 ± 0.35
Secreted frizzled‐related protein 3	0.92 ± 0.69	0.79 ± 0.66	1.03 ± 0.67
Ephrin type‐B receptor 6	3.43 ± 0.35	3.38 ± 0.32	3.42 ± 0.45
RGM domain family member B	5.14 ± 0.44	5.14 ± 0.43	5.21 ± 0.39
Sialoadhesin	4.05 ± 0.58	3.96 ± 0.58	3.99 ± 0.63
Contactin‐5	5.41 ± 0.44	5.36 ± 0.44	5.39 ± 0.36
Disintegrin and metalloproteinase domain‐containing protein 22	4.29 ± 0.44	4.20 ± 0.44	4.21 ± 0.45
C‐type lectin domain family 1 member B	11.41 ± 0.30	10.98 ± 0.38^a^	10.83 ± 0.29^a^
Disintegrin and metalloproteinase domain‐containing protein 23	3.11 ± 0.52	3.01 ± 0.48	3.01 ± 0.60
Matrilin‐3	1.74 ± 0.33	1.67 ± 0.28	1.72 ± 0.36
R‐spondin‐1	1.43 ± 0.44	1.28 ± 0.45	1.37 ± 0.53
Hydroxyacylglutathione hydrolase, mitochondrial	1.39 ± 1.74	1.37 ± 1.48	1.00 ± 0.97
Latexin	1.36 ± 1.46	1.26 ± 1.01	0.97 ± 0.25
Galectin‐8	6.13 ± 0.33	5.85 ± 0.31^a^	5.69 ± 0.33^a^
Brevican core protein	5.19 ± 0.34	5.21 ± 0.39	5.17 ± 0.37
Layilin	4.66 ± 0.41	4.57 ± 0.37	4.55 ± 0.56
Neprilysin	1.64 ± 0.74	1.49 ± 0.70	1.63 ± 0.79
Growth/differentiation factor 8	3.35 ± 0.60	3.29 ± 0.60	3.31 ± 0.56
Thy‐1 membrane glycoprotein	8.82 ± 0.22	8.80 ± 0.20	8.74 ± 0.22
WAP, Kazal, immunoglobulin, Kunitz and NTR domain‐containing protein 1	2.57 ± 0.54	2.57 ± 0.54	2.60 ± 0.53
Transmembrane protease serine 5	2.74 ± 0.51	2.66 ± 0.47	2.75 ± 0.45
Cadherin‐3	6.23 ± 0.49	6.10 ± 0.61	6.23 ± 0.50
GDNF family receptor alpha‐1	5.43 ± 0.30	5.35 ± 0.32	5.39 ± 0.33
Granulocyte‐macrophage colony‐stimulating factor receptor subunit alpha	5.20 ± 0.81	5.14 ± 0.78^a^	5.07 ± 0.86
Beta‐nerve growth factor	0.50 ± 0.28	0.44 ± 0.28	0.41 ± 0.31
Scavenger receptor class A member 5	6.65 ± 0.26	6.63 ± 0.33	6.55 ± 0.29
OX‐2 membrane glycoprotein	5.44 ± 0.39	5.41 ± 0.37	5.46 ± 0.42
BDNF/NT‐3 growth factors receptor	4.49 ± 0.26	4.44 ± 0.27	4.49 ± 0.26
Granzyme A	3.98 ± 0.48	3.81 ± 0.48^a^	3.83 ± 0.49
Granulocyte Colony‐Stimulating Factor	1.57 ± 0.50	1.57 ± 0.56	1.57 ± 0.70
Draxin	1.69 ± 0.48	1.76 ± 0.47	1.75 ± 0.64
Scavenger receptor class F member 2	4.92 ± 0.33	4.88 ± 0.33	4.88 ± 0.32
GDNF family receptor alpha‐3	3.75 ± 0.35	3.67 ± 0.37	3.73 ± 0.44
Poliovirus receptor	7.11 ± 0.36	7.05 ± 0.34	7.11 ± 0.36
Tumor necrosis factor receptor superfamily member 12A	5.31 ± 0.57	5.28 ± 0.51	5.29 ± 0.51
Serine/threonine‐protein kinase receptor R3	6.94 ± 0.29	6.82 ± 0.25	6.79 ± 0.35
Leucine‐rich repeat transmembrane protein FLRT2	1.32 ± 0.34	1.26 ± 0.28	1.26 ± 0.43
Carboxypeptidase M	6.08 ± 0.27	6.03 ± 0.21	6.08 ± 0.19
C‐type lectin domain family 10 member A	3.08 ± 0.42	3.11 ± 0.48	3.04 ± 0.48
Glypican‐5	4.33 ± 0.69	4.22 ± 0.62^a^	4.18 ± 0.59
Bone morphogenetic protein 4	1.95 ± 0.46	2.13 ± 0.47^a^	2.18 ± 0.53
Fc receptor‐like protein 2	4.45 ± 0.41	4.40 ± 0.35	4.41 ± 0.40
MAM domain‐containing glycosylphosphatidylinositol anchor protein 1	4.14 ± 0.69	4.14 ± 0.62	4.15 ± 0.74
Interleukin‐5 receptor subunit alpha	2.34 ± 0.58	2.18 ± 0.64	2.32 ± 0.64
Platelet‐derived growth factor receptor alpha	3.68 ± 0.36	3.56 ± 0.37^a^	3.57 ± 0.34
Dipeptidyl peptidase 1	4.01 ± 0.27	3.94 ± 0.32	3.87 ± 0.37
Cadherin‐6	3.89 ± 0.24	3.70 ± 0.84	3.88 ± 0.26
Epithelial discoidin domain‐containing receptor 1	5.41 ± 0.26	5.37 ± 0.25	5.39 ± 0.31
Junctional adhesion molecule B	7.49 ± 0.36	7.45 ± 0.35	7.41 ± 0.44
Cathepsin S	5.26 ± 0.22	5.17 ± 0.25^a^	5.16 ± 0.25
Neutral ceramidase	2.36 ± 0.65	2.25 ± 0.55	2.36 ± 0.66
N‐acylethanolamine‐hydrolyzing acid amidase	2.80 ± 0.32	2.63 ± 0.35	2.62 ± 0.49
NKG2D ligand 2	2.90 ± 0.45	2.79 ± 0.43	2.76 ± 0.53
Plexin‐B1	6.82 ± 0.96	6.27 ± 1.07^a^	6.55 ± 1.11
Tumor necrosis factor receptor superfamily member 21	8.54 ± 0.28	8.49 ± 0.29	8.51 ± 0.27
CMRF35‐like molecule 1	4.00 ± 0.60	3.85 ± 0.49^a^	3.79 ± 0.60
Testican‐1	2.04 ± 0.30	1.92 ± 0.30	2.07 ± 0.38
Interleukin‐12 subunit beta, Interleukin‐12 subunit alpha	7.46 ± 0.60	7.39 ± 0.55	7.46 ± 0.68
Dickkopf‐related protein 4	1.99 ± 0.53	1.92 ± 0.52	1.86 ± 0.63
Tumor necrosis factor receptor superfamily member 27	3.07 ± 0.44	3.05 ± 0.40	3.01 ± 0.42
Linker for activation of T‐cells family member 1	3.33 ± 0.37	3.19 ± 0.42	3.10 ± 0.45
NT‐3 growth factor receptor	6.25 ± 0.34	6.23 ± 0.32	6.26 ± 0.36
Leukocyte‐associated immunoglobulin‐like receptor 2	3.35 ± 0.82	3.35 ± 0.82	3.35 ± 0.91
Mesencephalic astrocyte‐derived neurotrophic factor	7.23 ± 0.22	6.86 ± 0.54^a^	6.56 ± 0.58^a^
Tenascin‐R	3.83 ± 0.49	3.70 ± 0.49	3.77 ± 0.53
Cell surface glycoprotein CD200 receptor 1	4.23 ± 0.39	4.19 ± 0.36	4.22 ± 0.50
Neuronal cell adhesion molecule	7.94 ± 0.18	7.95 ± 0.18	7.96 ± 0.20
Kynureninase	4.90 ± 0.60	4.91 ± 0.59	4.93 ± 0.62

a Indicates significant difference (*p* < .001), compared to fasting values.

## 4 DISCUSSION

To the best of our knowledge, this is the first study to evaluate the effect of food intake on plasma proteins measured by the Proseek Multiplex Neurology I kit. This investigation showed that 14 of the 92 investigated biomarkers were affected by food intake. The changes were, however, very modest. Only one biomarker showed a difference more than 10% due to food intake, the changes were on average about 5%. The observed effects of food intake are approximately half of the intra‐assay and inter‐assay variation which are about 10% for most of the biomarkers. This suggests that the need to standardize food intake is generally not necessary when using this kit (Proseek Multiplex Neurology I). There was, however, one exception. The biggest change was observed for Plexin‐B3 120 min after food intake (12%). It could be difficult in the acute setting to standardize food intake, but our finding clearly suggests that this should be done when investigating this biomarker.

Recent technological advances make it possible to measure multiple plasma proteins simultaneously. In this study, the Proseek Multiplex Neurology I kit was used to simultaneously measure 92 plasma proteins. These have been selected because of importance in neurological panorama. According to the manufacturer, 19 of the biomarkers are labeled as axon guidance, 19 exploratory (immunology, development and metabolism), 16 neural development, 13 exploratory (cellular regulation), 10 neurological disease associated, 8 neurology miscellaneous, and 7 synaptic function. Our investigation adds methodological information in this developing field.

We observed most of the changes 30 min after food intake. A total of 13 biomarkers decreased, and one increased, 30 min after food intake. This could most probably be due to the changes in hemodynamics. We have previously reported a 20% increase in stroke volume and a 28% increase in cardiac output 30 min after food intake in this cohort (Dencker et al., 2011). It could also be due to the fact that food intake changes several endocrinological pathways such as altered secretions of appetite and metabolic regulatory hormones such as insulin, ghrelin, glucagon, glucagon‐like peptide 1, as well as immune response and cellular metabolism due to the increased availability of nutrients (Hlebowicz et al., 2011a; Hlebowicz et al., 2011b; Stensel, 2010).

We are aware of only one study that has evaluated the impact of food intake on one of the biomarker (Beta‐nerve growth factor) in this study. Jahn and coworkers evaluated the effect of food intake on Beta‐nerve growth factor (Jahn et al., 2016). This study evaluated the levels before and 240 min after a high‐fat breakfast in 16 healthy and 18 metabolic syndrome subjects. Their findings were concordant with ours.

Our secondary aim was to investigate if any of the biomarkers were correlated with BMI. Inflammation has been shown to be correlated with obesity and with a variety of neurological disorders (Larsson et al., 2015), and several biomarkers in Proseek Multiplex Neurology I kit are in various ways immunological. This turned out to be very much a dead end, as only Kynureninase was significantly correlated with BMI of all the 92 biomarkers. This is perhaps not surprising given the fact that young, presumably healthy, subjects with a normal body composition participated in this investigation.

There are some limitations that should be addressed. The present investigation showed the effect of food intake in only young healthy Caucasian subjects. Studies are warranted in older healthy subjects from different ethnic groups and in patients with various neurological diseases to determine whether the findings in the present investigation are reproducible in such populations. In addition, future investigations are warranted to evaluate the effect of different diets such as high or low fat.

## 5 CONCLUSION

The present investigation shows that the effect of food intake is very modest on many neurological biomarkers. Timing of blood sampling in relation to food intake, therefore, appears not to be a major concern. There is, however, one exception. Caution concerning food intake should be observed when investigating Plexin‐B3. Of all the 92 neurological biomarkers only Kynureninase was significantly correlated with BMI.

## CONFLICT OF INTEREST

The authors declare that they have no conflicts of interest.
